# The UCSC Genome Browser database: 2026 update

**DOI:** 10.1093/nar/gkaf1250

**Published:** 2025-11-18

**Authors:** Jonathan Casper, Matthew L Speir, Brian J Raney, Gerardo Perez, Luis R Nassar, Christopher M Lee, Angie S Hinrichs, Jairo Navarro Gonzalez, Clay Fischer, Mark Diekhans, Hiram Clawson, Anna Benet-Pages, Galt P Barber, Charles J Vaske, Marijke J van Baren, Karen Wang, Yesenia Joanna Puga Rodriguez, Jaidan Ashlyn Jenkins-Kiefer, Megna Chalamala, David Haussler, William James Kent, Maximilian Haeussler

**Affiliations:** Genomics Institute, University of California Santa Cruz, Santa Cruz, CA 95064, United States; Genomics Institute, University of California Santa Cruz, Santa Cruz, CA 95064, United States; Genomics Institute, University of California Santa Cruz, Santa Cruz, CA 95064, United States; Genomics Institute, University of California Santa Cruz, Santa Cruz, CA 95064, United States; Genomics Institute, University of California Santa Cruz, Santa Cruz, CA 95064, United States; Genomics Institute, University of California Santa Cruz, Santa Cruz, CA 95064, United States; Genomics Institute, University of California Santa Cruz, Santa Cruz, CA 95064, United States; Genomics Institute, University of California Santa Cruz, Santa Cruz, CA 95064, United States; Genomics Institute, University of California Santa Cruz, Santa Cruz, CA 95064, United States; Genomics Institute, University of California Santa Cruz, Santa Cruz, CA 95064, United States; Genomics Institute, University of California Santa Cruz, Santa Cruz, CA 95064, United States; Institute of Neurogenomics, Helmholtz Zentrum Munchen GmbH—German Research Center for Environmental Health, 85764 Neuherberg, Germany; Medical Genetics Center (Medizinisch Genetisches Zentrum), Munich 80335, Germany; Genomics Institute, University of California Santa Cruz, Santa Cruz, CA 95064, United States; Genomics Institute, University of California Santa Cruz, Santa Cruz, CA 95064, United States; Genomics Institute, University of California Santa Cruz, Santa Cruz, CA 95064, United States; Genomics Institute, University of California Santa Cruz, Santa Cruz, CA 95064, United States; Genomics Institute, University of California Santa Cruz, Santa Cruz, CA 95064, United States; Genomics Institute, University of California Santa Cruz, Santa Cruz, CA 95064, United States; Genomics Institute, University of California Santa Cruz, Santa Cruz, CA 95064, United States; Genomics Institute, University of California Santa Cruz, Santa Cruz, CA 95064, United States; Genomics Institute, University of California Santa Cruz, Santa Cruz, CA 95064, United States; Genomics Institute, University of California Santa Cruz, Santa Cruz, CA 95064, United States

## Abstract

Now in its 25th year of operation, the UCSC Genome Browser (https://genome.ucsc.edu) provides a central location for researchers around the world to display and compare annotations on assembled genomes. Highlighted updates include a positional heatmap display, used to show data on functional consequences of mutation from MaveDB; QuickLift, a tool to copy annotation data seen on one assembly for display on another related assembly; and HubSpace, an initiative to simplify the process of creating and using track hubs by providing each user account with dedicated storage on UCSC’s infrastructure.

## Graphical Abstract

**Graphical Abstract:**
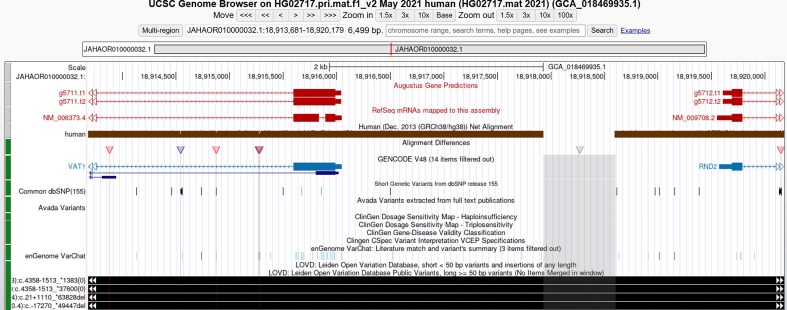

## Introduction

The UCSC Genome Browser (“the Browser”) is a web-based resource for the visualization and automated retrieval of genomic data and annotations that serves thousands of users per day. The Browser provides support to the community primarily via an interactive display of genomic regions and a REST API for access to most hosted data. It also includes tools for users to load and visualize their own annotation data alongside those provided by UCSC and to share those visualizations with other users. All data provided by UCSC are also available for direct download, apart from a small number of tracks under special restriction by the original data providers.

The primary unit of organization within the Browser is the assembly, which describes the baseline sequence and set of regions present within a genome. Including those within the GenArk assembly hub system [1], UCSC now provides a display for over 28 000 genome assemblies. Annotation data for each assembly are collected in “tracks,” each of which has a particular data format and topic of relevance. Some tracks are fixed and unchanging, such as a plot of GC content across the assembly, while others are periodically updated by UCSC, such as gene annotations. Users are also able to load their own annotation data for display alongside UCSC’s tracks using the “custom track” and “track hub” features. Custom tracks are intended for quick visualization of small datasets, where all data can be quickly uploaded to UCSC. Track hubs provide more structure, allow use of a wider variety of the Browser’s features, and are designed to be stored and accessed via independent web servers. UCSC curates a selection of documented community-provided hubs on its Public Hubs portal, and many more hubs are available from the EBI Track Hub Registry (https://trackhubregistry.org). The large majority of users interact with the GRCh37/hg19 and GRCh38/hg38 human genome assemblies, and as a result those assemblies contain orders of magnitude more tracks and track hubs than the rest; they also receive the most attention from UCSC when adding and updating annotations.

## Updates to genomes and annotations

The past year has seen the release of several new tracks and track displays, including an updated display for truncating mutations in our ClinVar [2] tracks, incorporation of multiplexed assays of variant effects (MAVEs) from MaveDB [3], and QuickLift, a method of converting annotation tracks from one assembly to another on the fly.

### Diamond display for protein-truncating mutations in ClinVar Variants

**Figure 1.**
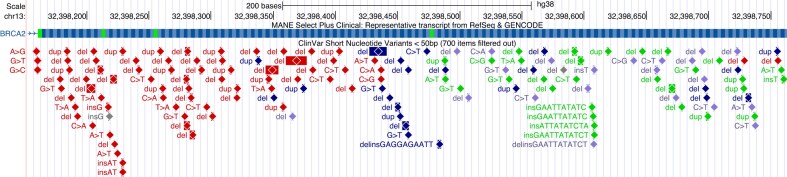
The ClinVar Variants track has been updated to indicate premature termination mutations with shaded diamonds. The diamond icons make it easier to identify pileups of these variants, which in turn suggest the presence of functionally important regions.

Inspired by the Decipher [4] transcript display, our existing track of variants from ClinVar on GRCh37/hg19 and GRCh38/hg38 has been updated to indicate the presence of premature termination codon mutations (Fig. 1). Protein regions with a high number of these mutations are often important to the function of the protein. The display now takes advantage of our decorators feature [5] to mark each of these variants with a colored diamond, which makes it easier to identify regions where those mutations pile up. The new display is available from the track configuration page and affects the following molecular consequence classes: splice acceptor variant, splice donor variant, frameshift variant, and nonsense.

### Multiplexed assays of variant effects from MaveDB

**Figure 2.**
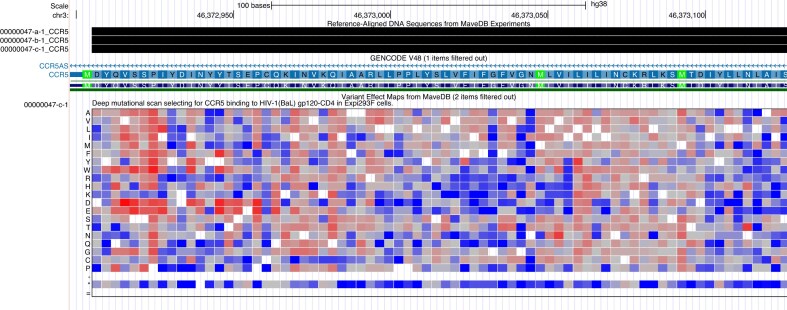
MaveDB heatmap data for point mutations within the CCR5 gene, indicating functional consequences when the reference codon is changed to a different peptide. Low scores, in blue, indicate decreased abundance, while high scores, in red, indicate increased abundance. Mouseover text provides details and the score(s) for each mutation.

The MaveDB project provides a growing amount of data on the consequences of mutational variation experiments, particularly MAVEs. These experiments systematically test the consequences of point mutations in functional elements. The results are of significant interest to both the research and clinical communities because of their breadth—they include many variants that are not described elsewhere. A subset of these data are now available in a heatmap track at UCSC, which shows consequences for expression of point mutations in shades of red and blue (Fig. 2). Users interested in taking advantage of this display type for their own data can consult our file formats page for details on the “heatmap” display style for bigBed files. The full format definition allows specification of a custom color scheme for each heatmap in the track; the colors in the MaveDB track were chosen to provide a close match to the colors used in that project.

**Figure 3.**
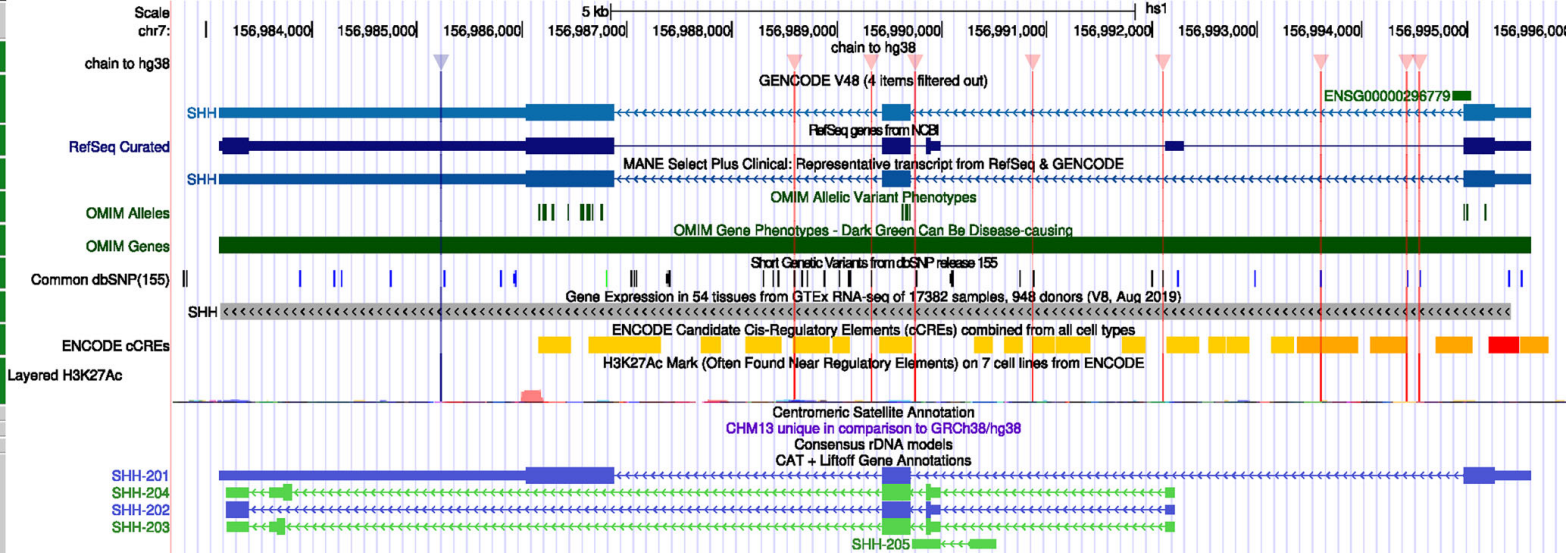
The new QuickLift tool was used to convert annotation tracks from the GRCh38/hg38 human assembly (marked in green on the left edge) to T2T-CHM13/hs1. Differences between the assemblies are marked on the display with vertical bars; a mouseover reveals that one difference is a single base sequence mismatch.

**Figure 4.**
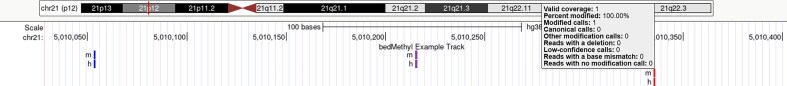
Epigenetic data loaded into a custom track using the newly supported bedMethyl format. Mouseover provides details about each modification.

**Figure 5.**
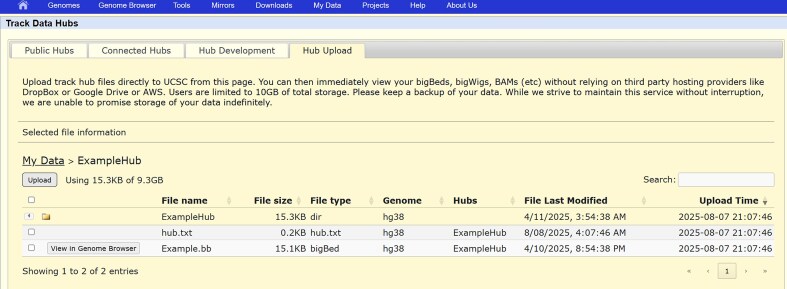
The new HubSpace interface allows each user to store up to 10GB of genomic data files directly on UCSC’s servers. Supported file formats are limited to those supported in track and assembly hubs.

### QuickLift

The new “QuickLift” tool permits dynamically lifting annotation from one assembly to another (Fig. 3). The vast majority of the Browser’s users spend their time exploring the human genome, split between the GRCh38/hg38 and GRCh37/hg19 assemblies (released in 2009 and 2013, respectively). While newer assemblies exist (e.g. the telomere-to-telomere release of T2T-CHM13/hs1 [6] in 2022), they have significantly fewer annotations and consequently are less in use. QuickLift addresses this bottleneck by allowing users to transpose data from one assembly onto another as part of an interactive browsing session. This facilitates research on newer human assemblies as well as the myriad genome assemblies available via our GenArk project, which provides basic browsers for over 28 000 genome assemblies from RefSeq [7] and GenBank [8]. QuickLift provides a much-needed path for augmenting those basic browsers with details from other sources.

The QuickLift feature is available (at time of writing) from the *View → In Other Genomes* item in the Browser’s menu bar. Start by visiting the assembly with the annotation of interest, then use *In Other Genomes* to select the desired destination and check the box for “QuickLift tracks.” QuickLift availability requires the presence of a liftOver alignment between the source and destination assemblies; such alignments can be created by UCSC upon request (https://genome.ucsc.edu/contacts.html), and we are working with Galaxy [9] to set up an automated pipeline for handling such requests in the future.

### Other data

A full list of tracks and assemblies released over the year can be found in our release log online at https://genome.ucsc.edu/goldenPath/releaseLog.html, with the announcements archived at https://genome.ucsc.edu/goldenPath/newsarch.html.

## User-contributed data options

While UCSC continues to add annotation tracks itself, the growth in the number of available genomes and assemblies has underscored the importance of supporting our users in loading their own data for display. Several features were added to the Browser this year to support users in loading, displaying, and sharing their own data in the Browser alongside UCSC-provided tracks.

### BedMethyl data

The Browser added a new track type this year for *bedMethyl* data files (Fig. 4), following the extended format described at https://nanoporetech.github.io/modkit/intro_pileup.html. Nanopore sequencing assays provide more information about methylation and other epigenetic states of nucleic acids. bedMethyl files provide a convenient method for transmitting and displaying this information in browsers, and the popular modKit (https://github.com/nanoporetech/modkit) software uses this format. More information on this track type and examples of its use can be found at https://genome.ucsc.edu/goldenPath/help/bedMethyl.html.

### GenArk contributed tracks

UCSC has begun encouraging research communities around the world to contribute annotation tracks to the GenArk assembly hub project. The GenArk project has already built nearly 30 000 baseline assembly hubs for organisms in GenBank and RefSeq, with many more on the horizon. This is tied to a corresponding explosion in the amount of data available from the research communities for those organisms, which UCSC is now working to integrate. We are already collaborating with TOGA [10] and the T2T consortium and are eager to expand that list. We invite labs to contact us (https://genome.ucsc.edu/contacts.html) if they have suitable data to share.

### HubSpace

The Browser also took steps this year to make it easier for researchers to store and display data in the Genome Browser either for their own use or as a preface to inclusion in GenArk. While we already allow users to link to their own data files on the web via the track hub and custom track features, it can be difficult to find hosting space that meets the associated needs for random access and storage space. The new HubSpace system at UCSC (see the “Hub Upload” tab at https://genome.ucsc.edu/cgi-bin/hgHubConnect) provides each user account with storage that is co-located with the Genome Browser servers and which can be used to store files associated with track and assembly hubs (Fig. 5). The exact amount of storage provided is still in flux as we match our capabilities against the community’s needs, but it is in the range of 10–20 GB for each account with more available upon request. While there is no charge for this service, we encourage users to maintain backups of all of their files.

### Future plans

Upcoming feature goals for the Browser focus on further facilitating users to display and share their own data. One such project is My Annotations, a system for easily creating, maintaining, and sharing a personal curated set of genomic variants with accompanying annotation. Management of this set would occur entirely within the Browser’s web interface, removing the need for manual file interactions. Another topic of interest is support for opening local annotation files from user systems and displaying them without the data leaving those systems. The current Browser tools for displaying user data all involve transmission to UCSC, which can violate HIPAA and related legal requirements. A method for keeping all user data on the user’s own system would solve this problem, permitting researchers to begin examining restricted data in the context of UCSC’s rich annotation sets.

## Data availibility

The UCSC Genome Browser website and APIs are freely available for all users. Most data files provided by UCSC are also freely available, and much of the Genome Browser source code is provided under the MIT license. See our License page (https://genome.ucsc.edu/license/) for full details. Files can be found on our download server at https://hgdownload.soe.ucsc.edu as well as at https://doi.org/10.5281/zenodo.17411096.

## References

[1] Clawson H , Lee BT, Raney BJ et al. GenArk: towards a million UCSC genome browsers. Genome Biol. 2023;24:217. 10.1186/s13059-023-03057-x. 37784172 PMC10544498

[2] Landrum MJ , Chitipiralla S, Kaur K et al. ClinVar: updates to support classifications of both germline and somatic variants. Nucleic Acids Res. 2025;53:D1313–21. 10.1093/nar/gkae1090. 39578691 PMC11701624

[3] Arbesfeld JA , Da EY, Stevenson JS et al. Mapping MAVE data for use in human genomics applications. Genome Biol. 2025;26:179. 10.1186/s13059-025-03647-x. 40563119 PMC12188674

[4] Foreman J , Perrett D, Mazaika E et al. DECIPHER: improving genetic diagnosis through dynamic integration of genomic and clinical Data. Annu Rev Genom Hum Genet. 2023;24:151–76. 10.1146/annurev-genom-102822-100509. PMC7615097 37285546

[5] Raney BJ , Barber GP, Benet-Pagès A et al. The UCSC Genome Browser database: 2024 update. Nucleic Acids Res. 2024;52:D1082–8. 10.1093/nar/gkad987. 37953330 PMC10767968

[6] Nurk S , Koren S, Rhie A et al. The complete sequence of a human genome. Science. 2022;376:44–53. 10.1126/science.abj6987. 35357919 PMC9186530

[7] Goldfarb T , Kodali VK, Pujar S et al. NCBI RefSeq: reference sequence standards through 25 years of curation and annotation. Nucleic Acids Res. 2025;53:D243–57. 10.1093/nar/gkae1038. 39526381 PMC11701664

[8] Clark K , Karsch-Mizrachi I, Lipman DJ et al. GenBank. Nucleic Acids Res. 2016;44:D67–72. 10.1093/nar/gkv1276. 26590407 PMC4702903

[9] Galaxy Community . The Galaxy platform for accessible, reproducible, and collaborative data analyses: 2024 update. Nucleic Acids Res. 2024;52:W83–94. 10.1093/nar/gkae410. 38769056 PMC11223835

[10] Kirilenko BM , Munegowda C, Osipova E et al. Integrating gene annotation with orthology inference at scale. Science. 2023;380:eabn3107. 10.1126/science.abn3107. 37104600 PMC10193443

